# Case of Poisening, from Swallowing Percussion Caps

**Published:** 1847-06

**Authors:** T. W. Foster

**Affiliations:** Keene, Jessamin County, Ky.


*Case of Poisening, from swallowing Percussion Caps.*—By T. W. Foster, M. D., of Keene, Jessamin County, Ky.

Not long since, I was called in great haste to attend an infant, æt. 14 months. Upon entering the room, I was informed by the parents that they had observed their child, about two hours previous to my visit, playing with a box of percussion caps, and they supposed she had swallowed some of them, as signs of acute suffering were exhibited soon after.

The little patient appeared to be sinking very fast. The eyes had a hollow, glazed appearance; there was great heat in the epigastric region, and coldness of the extremities; there had been eight or nine discharges from the bowels in an hour, and her general aspect denoted approaching collapse. Before my arrival free emesis had been produced by some domestic remedy, yet I continued the vomiting by administering ipecac. and large draughts of warm water, (of which the patient greedily drank,) with the hope of discharging at least a portion of the offending matters. The discharges became so debilitating, however, that I threw up an injection of eight drops of laudanum, suspended in starch mucilage, and immediately afterward gave a large dose of calcined magnesia. An alkaline purgative was selected for the purpose of neutralizing any acid which might be found in the stomach or intestines, and thus prevent any chemical change in the copper. In the course of an hour, the child became perfectly composed, and fell into a pleasant slumber, though it had previously suffered excuciating pain, attended with spasms. Dr. Spilman, the family physician, now took charge of the case, and applied counter-irritation to the abdomen. On the next day four caps were discovered in the fœcal matter, which were found to be devoid of their fulminating powder. The child is now enjoying very good health.

